# Effectiveness of Single Nucleotide Polymorphism Markers in Genotyping Germplasm Collections of *Coffea canephora* Using KASP Assay

**DOI:** 10.3389/fpls.2020.612593

**Published:** 2021-01-25

**Authors:** Abraham Akpertey, Francis K. Padi, Lyndel Meinhardt, Dapeng Zhang

**Affiliations:** ^1^Cocoa Research Institute of Ghana, New Tafo Akyem, Ghana; ^2^Sustainable Perennial Crops Laboratory, Agricultural Research Service, United States Department of Agriculture, Beltsville, MD, United States

**Keywords:** conservation, DNA fingerprinting, genetic diversity, Genebank, West Africa

## Abstract

Accurate genotype identification is imperative for effective use of *Coffea canephora* L. germplasm to breed new varieties with tolerance or resistance to biotic and abiotic stresses (including moisture stress and pest and disease stresses such as coffee berry borer and rust) and for high yield and improved cup quality. The present study validated 192 published single nucleotide polymorphism (SNP) markers and selected a panel of 120 loci to examine parentage and labeling errors, genetic diversity, and population structure in 400 *C. canephora* accessions assembled from different coffee-producing countries and planted in a field gene bank in Ghana. Of the 400 genotypes analyzed, both synonymous (trees with same SNP profiles but different names, 12.8%) and homonymous (trees with same name but different SNP profiles, 5.8%) mislabeling were identified. Parentage analysis showed that 33.3% of the progenies derived from controlled crossing and 0% of the progenies derived from an open pollinated biclonal seed garden had parentage (both parents) corresponding to breeder records. The results suggest mislabeling of the mother trees used in seed gardens and pollen contamination from unwanted paternal parents. After removing the duplicated accessions, Bayesian clustering analysis partitioned the 270 unique genotypes into two main populations. Analysis of molecular variance (AMOVA) showed that the between-population variation accounts for 41% of the total molecular variation and the genetic divergence was highly significant (*F*st = 0.256; *P* < 0.001). Taken together, our results demonstrate the effectiveness of using the selected SNP panel in gene bank management, varietal identification, seed garden management, nursery verification, and coffee bean authentication for *C. canephora* breeding programs.

## Introduction

Coffee belongs to the Rubiaceae family and the genus *Coffea*. Of the approximately 124 species in the *Coffea* genus (Davis, 2011), *C. arabica* (generally called Arabica) and *C. canephora* Pierre ex A. Froehner (generally called Robusta) are cultivated the most globally. The two species make up approximately two thirds and one third of the total global production, respectively (ICO, 2020). *C. canephora* coffee is a diploid species (2n = 2x = 22) and mainly self-incompatible (allogamous) compared with Arabica coffee, which is an allotetraploid (2n = 4x = 44) and largely self-compatible (autogamous). *C. canephora* has increased productivity and higher caffeine content than *C. arabica*. Additionally, *C. canephora* tends to grow better at lower altitude and is more resilient to pests, diseases, and drought compared with *C. arabica* (DaMatta et al., 2007).

Genetic diversity studies have revealed two main genetic groups (Congolese and Guinean groups) in *C. canephora* (Berthaud, 1986). The Congolese group originates mainly from the Democratic Republic of Congo (DRC), Cameroon, and Central Africa Republic (CAR), whereas the Guinean group originates from Guinea and the Ivory Coast (Berthaud, 1986). A substructure with two subdivisions in the Congolese group was suggested by Montagnon et al. (1992). Similarly, Dussert et al. (1999) proposed two additional groups, B and C, to the Congolese group. Within the Guinea–Congo zone, five groups were separated by Gomez et al. (2009) in their *C. canephora* genetic diversity studies. Within the five groups, Guinean *C. canephora*, which is separated geographically by the Dahomey Gap, was identified as diversity group D, whereas diversity groups A, B, C, and E were found to be in the geographical area of DRC, Cameroon, and CAR (Gomez et al., 2009). A possible new diversity group within *C. canephora* was identified by Musoli et al. (2009) in their study, which found substantial genetic structure in *C. canephora* samples from Uganda that were genetically dissimilar from earlier identified diversity groups of *C. canephora*.

The main coffee type cultivated in Ghana is *C. canephora*, largely due to ecological adaptation reasons. At present, the exact genetic diversity group (Congolese or Guinean group) to which the CRIG *C. canephora* accessions belong is unknown. Although the genetic base is unknown, the collection at CRIG is made up of introductions made in 1977 and 1992 from the Cote d’Ivoire (Martinson et al., 1982; Anim-Kwapong and Adomako, 2010) and Togo (Anim-Kwapong and Adomako, 2010), respectively. Also, the *C. canephora* germplasm collection at CRIG consists of local collections made in 2009 from the defunct Ghana Cocoa Board plantations situated in the Western Region of the country. The coffee trees in the defunct Ghana Cocoa Board plantations are thought to have originated from Uganda and Tanzania (Anim-Kwapong et al., 2010). A more recent inclusion in 2012 to the coffee germplasm collection at CRIG is 72 introductions from Togo that are currently being evaluated for yield and other agronomic traits (Anim-Kwapong et al., 2013). Furthermore, in 2016, two *C. canephora* varieties were introduced as seeds from Vietnam and are currently being evaluated in the field for yield and other agronomic traits.

The *C. canephora* germplasm collection in CRIG is preserved as trees in the field gene bank because coffee seeds are recalcitrant and are unsuitable to be stored by traditional seed storage methods. Typically, each germplasm accession is represented by multiple trees, which are propagated by cuttings (for clonal accessions). Gene bank activities may be affected by improper identification, redundancy, and other errors because the trees in the germplasm plots were collected, exchanged, or otherwise obtained at different times with limited passport data on their correct identity. Moreover, limited knowledge on the level of genetic diversity of the *C. canephora* germplasm hinders their effective utilization in a breeding program (Akpertey et al., 2019). Previous efforts on genotype identification and diversity assessment in the coffee germplasm collections in Ghana have relied largely on morphological characters (Anim-Kwapong et al., 2010; Akpertey et al., 2019), which is limited because the environment tends to have a significant effect on phenotypic expression (Souza et al., 2013). Precise identification of plants in any germplasm collection is important to facilitate its management and utilization. A cost-effective molecular marker system that is highly reliable is, thus, needed to assist coffee germplasm management.

Different molecular markers, especially simple sequence repeat (SSRs), also known as microsatellite markers, have been developed and are currently available to the coffee research community (Combes et al., 2000; Rovelli et al., 2000; Moncada and McCouch, 2004; Cubry et al., 2008; Missio et al., 2009; Vieira et al., 2010; Ferrão et al., 2015). The utility of these markers has been shown for different research purposes in *C. canephora*, including determination of genetic diversity and germplasm characterization (Prakash et al., 2005; Hendre et al., 2008; Silvestrini et al., 2008; Musoli et al., 2009; Ng’homa et al., 2017), development of genetic linkage maps (Paillard et al., 1996; Lashermes et al., 2001; Lefebvre-Pautigny et al., 2010), and identification of QTLs through linkage mapping (Leroy et al., 2011; Merot-L’Anthoene et al., 2014; Achar et al., 2015). Although SSR markers are highly useful in assisting *C. canephora* germplasm management, adaptation of SSR markers in high throughput genotyping has yet to be realized, which affects the cost-effectiveness of using SSR markers in large-scale genotyping (Zhang et al., 2020).

Single nucleotide polymorphism (SNP) markers are the most abundant marker type and suitable for analysis on a wide genomic scale (Ghosh et al., 2002; Rafalski, 2002) in breeding programs. Compared with microsatellite markers, SNPs are more cost-effective and amenable to high-throughput automation (Gupta et al., 2001) yet are less polymorphic (Ellegren, 2004). Due to the abundance and easy scoring of SNPs over other marker systems, they have become the markers of choice for genetic research in *C. canephora*, such as diversity analysis and molecular characterization of germplasm (Garavito et al., 2016; Bikila et al., 2017; Anagbogu et al., 2019), marker-assisted selection (Alkimim et al., 2020), and genotype identification (Zhou et al., 2016; Zhang et al., 2020). Recently, Merot-L’Anthoene et al. (2019) reported the development of an 8.5K SNP array, of which 5530 were discovered from *C. canephora*. This array, therefore, provides ample candidate SNPs for array-based genotyping that can be used for a wide choice of applications, including assessment of genetic diversity, detection of mislabeling, parentage analysis in breeding and seed programs, and genetic description of selections to facilitate variety development (Zhang et al., 2020).

The objective of the present pilot study was to assess a small subset of SNP markers for their effectiveness in germplasm identification, diversity analysis, and parentage verification in 400 *C. canephora* germplasm accessions assembled from different sources over different time periods at the Cocoa Research Institute of Ghana. Meeting the set objectives should provide us with a better assessment of the implications of genetic integrity in a *C. canephora* breeding program.

## Materials and Methods

### *C. canephora* Sample Analysis and SNP Genotyping

A total of 400 *C. canephora* trees representing 294 *C. canephora* clones or families (Table 1) assembled over different time scales of the coffee improvement program at the Cocoa Research Institute of Ghana (CRIG) were selected for genotyping. The 400 trees are made up of 17 introductions from Cameroon, 78 from Cote d’Ivoire (CNRA), 120 hybrid and clone selections made at CRIG, 54 local collections from Ghana, 68 from Togo, 30 from unknown sources, and 33 from Vietnam. A detailed list of genotypes and their origins/sources is presented in Supplementary Table S1. Passport data for most of the accessions was not available; therefore, we relied solely on historical records at CRIG to assign the various *C. canephora* materials based on country of origin. Three families were selected for the study of parentage analysis. Of the three families, two (H234 × H207 and B2 × E139) were derived from controlled crossing (full-sib), whereas one (E139 × C134) was derived from an open pollinated biclonal seed garden (half-sib) located at the experimental fields of CRIG, Tafo (Latitude 06° 13′N, Longitude 0° 22′W). Each tree was tagged with a plastic label, and young leaves were collected into labeled paper envelopes prior to leaf disk punching and further analysis.

**TABLE 1:** Source of 400 *C. canephora* genotypes analyzed from CRIG germplasm collection.

**Source of sample**	**Number of trees selected**	**Location of trees sampled**	**Total number of clones or families**
Collection of earlier *C. canephora* introductions to CRIG, Coffee museum at CRIG, Tafo	38	Germplasm plots of the Cocoa Research Institute of Ghana, Tafo (06°13′ N, 0°22′ W)	38
Evaluation of local clones collected from COCOBOD plantations in seven localities in Ghana, Plot OX4 at CRIG, Tafo	57	”	57
Evaluation of *C. canephora* introduction from Togo, Plot OX5 at CRIG, Tafo	57	”	57
Evaluation observational trial of recent introduction from Vietnam, Plot NX1 at CRIG, Tafo	27	”	27
Evaluation of half-sib families of *C. canephora*, Plot HX8 at CRIG, Tafo	26	”	6
Evaluation of working collection of *C. canephora* clones selected at CRIG, Plot DX3B at CRIG, Tafo	144	”	72
Development of hybrid varieties of *C. canephora* at CRIG, plot DX2 at CRIG, Tafo	12	”	2
Evaluation of high-yielding *C. canephora* clones selected from a number of hybrid trials at CRIG, plot UX5 at CRIG, Tafo	30	”	30
Biclonal seed garden plot at Tafo, coffee seed garden at CRIG, Tafo	4	Seed garden plot of the Cocoa Research Institute of Ghana, Tafo (06°13′ N, 0°22′ W)	2
Biclonal seed garden plot at Afosu, coffee seed garden at CRIG, Afosu	4	Seed garden plot of the Cocoa Research Institute of Ghana, Afosu Sub-station (6°22′ N, 0°59′ W)	2
Collection of different *C. canephora* types at Bunso, Coffee museum III at CRIG, Bunso	1	Germplasm plot of the Cocoa Research Institute of Ghana, Bunso Sub-station (41°33′ N, 14°33′ E)	1
**Total**	**400**		**294**

A total of 192 SNP markers were selected from the 8.5K array published by Merot-L’Anthoene et al. (2019). From SNPs mapped on the 11 chromosomes, approximately 3.5% were randomly selected using the RAND function in Excel 2016. The map positions and flanking sequences (used to design the primers) of the 192 candidate SNPs are presented in Supplementary Table S2. The 192 candidate SNPs were submitted to LGC Biosearch Technologies for a KASPar assay design based on the SNP locus-flanking sequences. Genomic DNA was extracted from young leaves using the sbeadex^TM^ mini plant kit. SNP genotyping was done using the Kompetitive allele-specific PCR (KASP chemistry) (Biosearch Technologies, Hoddesdon, Hertfordshire, United Kingdom). The KASPar^TM^ Genotyping System from LGC Biosearch Technologies is a competitive allele-specific dual FRET-based assay for SNP genotyping (Cuppen, 2007). The genotyping followed an in-house LGC protocol, and the genotype calling was conducted with SNPviewer (LGC Biosearch Technologies, Hoddesdon, United Kingdom).

### Assessment of Families for Agronomic Performance

Three families derived from two different plantings of ongoing trials were sampled for parentage analyses. Two families (H234 × H207 and B2 × E139) were obtained through controlled manual crossing, whereas the third (E139 × C134) was derived from an open-pollinated biclonal seed garden. Data on stem diameter growth of trees of the various families in these trials commenced 3 months after planting. Stem diameter (mm) was measured at 15 cm above ground level using electronic calipers. Growth data (during the prebearing phase) and yield data on the family H234 × H207 are presented in detail to show how mislabeling can negatively affect agronomic performance of coffee trees.

### Data Analysis

Raw data for the SNP loci and sample calls were organized in Microsoft Excel 2007. Quality control was performed using the Quality Assurance Module from SNP Variation Suite version 8 (SVS8; Golden Helix Inc., Bozeman, Montana). Any SNP having more than a 10% no-call rate was removed from the data set. SNPs that were in linkage disequilibrium (LD) with each other at *r*^2^ ≥ 0.5 were also removed. The filtered data were then used in subsequent analysis.

To determine the informativeness of the SNP markers, the GenAlEx 6.501 program (Peakall and Smouse, 2012) was used to compute key descriptive statistics, such as Shannon’s information index (*I*s), observed heterozygosity (*H*_O_), expected heterozygosity (H_E_), and inbreeding coefficient (*F*_IS_). The program CERVUS was used to compute the polymorphic information content (PIC) of each SNP and the probability of identity (PID) of the SNPs. The PID among siblings (PID-sib) is defined as the probability that two sibling individuals drawn at random from a population have the same mutilocus genotype (Waits et al., 2001). For genotype identification, pairwise multilocus matching was applied among individual samples in CERVUS using the Identity Analysis module. Sequences of any two individuals that were fully matched at all SNP loci were declared same genotype (or clones). Filtering and discarding of duplicates after the multilocus matching analysis resulted in 270 unique genotypes that were used for further analysis. Trees with the same SNP profiles but different names were declared synonymous. Similarly, trees with the same name but different SNP profiles were declared homonymous.

Pairwise relatedness (*r*) based on the method of Lynch and Ritland (1999) with the 2x option was estimated using GenAlEx 6.501 to detect kinship within *C. canephora* genotypes of the same group. The Lynch and Ritland method (2x option) assumes values from −1 (completely unrelated individuals) to 1 (identical twins). Values close to 0.5 indicate a full-sibling relationship, and a value of 0.25 indicates a half-sibling relationship. To calculate pairwise relatedness among the various *C. canephora* groups, we used the allele frequency of the total population of assembled genotypes with 9999 permutations in the pairwise relatedness function in the GenAlEx 6.501 program.

Parentage analysis was conducted using the software CERVUS 3.0 (Marshall et al., 1998; Kalinowski et al., 2007) to identify parent pairs for each of the trees derived from manual pollination or open-pollinated biclonal seed gardens. The list of candidate parents comprised all 270 unique genotypes identified after the multilocus matching analysis. In CERVUS, an error rate of 0.01% was used as the proportion of mistyped loci. In the parentage analysis, simulations were run for 20,000 cycles, assuming that 90% of the parents were sampled, and a total loci type rate of 95%. Critical likelihood values (LOD scores) of 95% (strict) and 80% (relaxed) confidence in assignments were obtained using simulations.

Bayesian clustering analysis in STRUCTURE v2.3.4 (Pritchard et al., 2000) was used to infer population structure. An admixture model was used, and the analyses were carried out without considering prior information about the genetic groups or geographic origins of the samples. Ten independent runs were assessed for each fixed number of clusters (*K* value) ranging from 1 to 15, each consisting of a burn-in of 100,000 iterations and 200,000 Markov chain Monte Carlo repetitions. The most likely number of clusters was detected using the methods of Evanno et al. (2005), and the computation was performed using the online program STRUCTURE HARVESTER (Earl and VonHoldt, 2012^1^). The program CLUMPP 1.1.2 (Jakobsson and Rosenberg, 2007) and DISTRUCT1.1 (Rosenberg, 2002) were used to visualize the result.

Genotypes were assigned to a cluster if the probability of membership was greater than 0.70. Analysis of molecular variance (*n* = 9999 permutations) and pairwise Fst were then used to test the results of STRUCTURE using GenAlEx 6.501 (Peakall and Smouse, 2006, 2012) and to determine the proportion of genetic variation that was attributed to differences between genetic clusters. To provide a complementary illustration of the genetic relationships among germplasm groups, a distance-based multivariate analysis was performed on the genetic clusters assigned by STRUCTURE analysis. Pairwise genetic distances were calculated based on the STRUCTURE assigned groups, and the genetic distance was used to conduct the principal coordinate analysis (PCoA) using a covariance matrix with data standardization option. The PCoA results are presented as two-axis PCO plots, and both plots axis 1 vs. 2 and axis 1 vs. 3 are presented separately.

## Results

### Genotyping Results and SNP Markers

Out of the 192 SNPs used to genotype the coffee accessions, a total of 187 SNPs successfully amplified (with a >90% overall amplification). Data filtering was further performed to remove SNPs in LD at *r*^2^ ≥ 0.5. After data filtering, a total of 120 SNPs were retained and used in further analysis. The genetic parameter estimates, including minor allele frequency (MAF), Is, H_O_, H_E_, PIC, and F_IS_, of the retained 120 SNPs are presented in Supplementary Table S3.

### Identification of Mislabeling

For genotype identification, accessions that were fully matched at the genotyped SNP loci were affirmed to be the same genotype (or clones). Synonymous and homonymous mislabeling among the *C. canephora* genotypes were identified following the pairwise multilocus matching analysis. The probability of two individuals having the same multilocus genotype (probability of identity among siblings) based on the 120 SNP loci was 1.8 × 10^–19^, which indicates that there is almost a null probability of finding two individuals with the same genotype in the population. The average PID-sib of the 120 SNP panel was 0.71 (Supplementary Table S4).

Of the 400 genotypes analyzed, the frequency of synonymous mislabeling (trees with the same SNP profiles but different names) was 12.8%, whereas the frequency of homonymous mislabeling (trees with the same name but different SNP profiles) was 5.8%. Based on breeders’ records, the synonymous mislabeling was mainly in two forms: (a) trees/clones have been mislabeled at CRIG in the breeding process over time, leading to duplications, and (b) the same clone was introduced from a particular country at different times with different names.

### Parentage and Pedigree Verification

Parentage analysis was conducted to identify parent pairs for each tree belonging to two families obtained through controlled crossing and one other family derived from an open-pollinated biclonal seed garden. Generally, among all 32 progeny trees analyzed from the three families, female parental clones were assigned at a high frequency and confidence in all families. However, of 12 progenies derived from controlled manual crossing, only four had parentage (both parents) corresponding to breeders’ records (Table 2). Six out of the remaining eight had female parents correctly identified. This observation points to pollen contamination being the principal cause of unintended parentage in hybrid progeny although mislabeling and/or extraneous seeds in the seed lot before sowing may also be implicated in wrong parentage of seed-derived coffee varieties. For the 20 progenies derived from an open pollinated biclonal seed garden (E139 × C134), only four had one parent corresponding to breeders’ records (Table 2). A number of putative male parents different from breeders’ records were found contributing to the parentage of the (E139 × C134) family. The detailed likelihood maternal and paternal parentage assignment for all 32 progeny trees is presented in Supplementary Table S5.

**TABLE 2:** Summary likelihood assignment of parentage of 32 *C. canephora* trees from three families based on 120 SNP markers.

**Family^a^**	**Number, *N***	**Female parent**			**Male parent**			
		***Nf***	**Pair LOD**	**Probable parent**	***Nm***	**Pair LOD**	**Probable parent^b^**	**Trio LOD**
B2 × E139	6	4	1.29–9.02	B2	0(3)	2.48–8.98	*E138*	13.11–45.68
H234 × H207	6	6	18.88-35.46	H234	4(2)	−3.00-2.95	H207	29.54–41.88
E139 × C134	20	3	13.49-22.61	*E138*	1(12)	Not applicable	*12 clones*^c^	7.92–49.23

*^*a*^Families B2 × E139 and H234 × H207 were derived through controlled crossing, whereas family E139 × C134 was obtained from the open-pollinated biclonal seed garden.*

*N refers to the number of individuals sampled for each family; *Nf* refers to the number of individuals that had the correct female parent; *Nm:* outside and inside brackets refer to the number of individuals that had the correct male parent in agreement with breeders’ records and the number of individuals with the probable male parent assigned by CERVUS, respectively; Pair LOD and Trio LOD denotes progeny–parent pair LOD score for probable parent matches.*

*^*b*^Probable parents in italics indicate pedigree that was in disagreement with breeders’ records of the parent used to generate the rest of the plants obtained from difference between the number of individuals sampled for each family (*N*) and number of individuals with the correct female (*Nf*) or male parent (*Nm*).*

*^*c*^A number of clones from nearby trials to the biclonal seed garden from which the E139 × C134 family was derived.*

### Effect of Mislabeling and Pollen Contamination on Agronomic Performance

**FIGURE 1:**
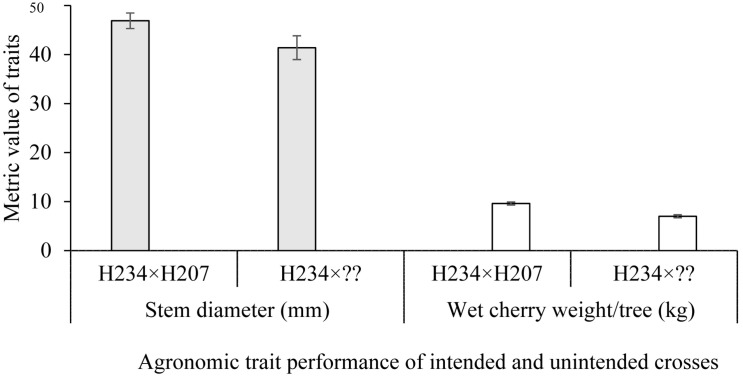
Stem diameter and weight of cherry per tree for a plot interplanted with progenies derived from an intended cross (H234 × H207; rightfully assigned by CERVUS and in agreement with breeder’s records) and an unintended cross (H234 × ??). ??, denotes mixed pollen from MMC26 and A129; identified by CERVUS in complete disagreement with breeder’s records). Stem diameter increment between 2010 and 2013 and average wet cherry weight from 2015 to 2018 were used, respectively for the analysis. Bar represents the standard error of the mean.

To demonstrate the effects of mislabeling and/or pollen contamination on agronomic performance of *C. canephora* trees, six progenies derived from the cross H234 × H207 were used. Of the six progenies, only four had the expected parentage of H234 × H207. Parentage analysis revealed two were of the pedigree H234 × MMC26 and H234 × A129 (Table 3). Differences in stem diameter growth and wet cherry weight per tree observed in the plot could be related to the effects of male parentage on progeny performance. Trees obtained from parent H207 (H234 × H207) were significantly (*P* < 0.05) larger in terms of stem diameter over a 4-year period and produced more berries (*P* < 0.05) during the matured stages of growth than those with either MMC26 or A129 as the male parent (Table 3 and Figure 1).

**TABLE 3:** Likelihood assignment of parentage from CERVUS and agronomic performance for 6 *C. canephora* trees obtained from a field plot interplanted with progenies obtained from crosses between H234 (female) and H207 (correct male) and a mixture of pollen (wrong) from two other males (MMC26) and (A129).

**Progeny ID**	**Female Parent (H234)**		**Male parent^a^**				**Parent-offspring**		**Agronomic trait performance**	
			**H207**		**??**				**Stem diameter (mm)**	**Wet cherry weight/tree (kg)**
	**Pair LOD**	**Conf**	**Pair LOD**	**Conf**	**Pair LOD**	**Conf**	**Trio LOD**	**Trio Conf**		
BP40_1	19.99	*	–3.11				31.27	*	46.0	8.8
BP40_2	26.92	*	–0.66				41.88	*	54.6	10.1
BP40_3	18.88	*	–3.00				34.25	*	40.5	10.7
BP40_6	13.94	*	2.95				31.87	*	46.6	8.9
BP40_4	35.46	*			5.20		33.18	*	45.1	7.2
BP40_5	23.07	*			2.25		29.54	*	37.7	6.9

^*a*^??: MMC26 was the assigned paternal parent for progeny BP40_4 and A129 was the assigned paternal parent for BP40_5. *Denotes parentage likelihood assignment at 95% confidence level.

### Population Structure and Relatedness in 270 Accessions With Unique SNP Profiles

**FIGURE 2:**
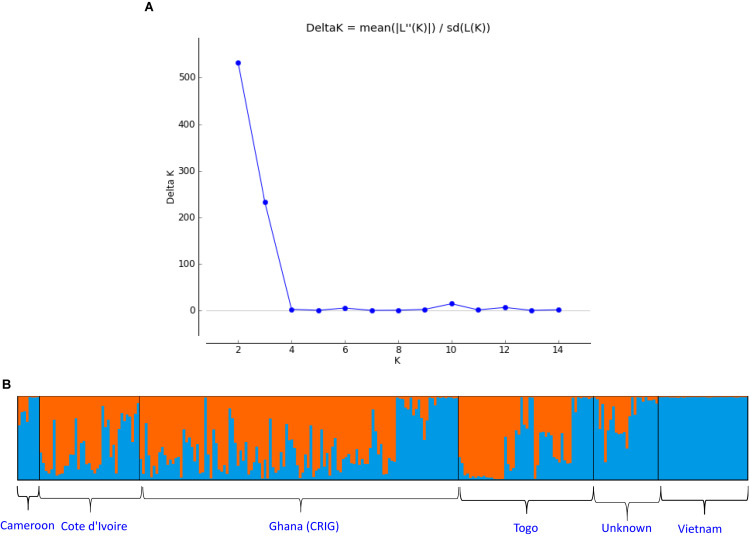
**(A)** Estimating number of subpopulations using delta *K* values for *K* ranging from 1 to 15 using the method proposed by Evanno et al. (2005). Delta K = mean(| L”(K)|)/sd(L(K)).**(B)** *Q*-plot showing clustering of 270 *C. canephora* genotypes based on analysis of genotypic data at 120 SNP loci using STRUCTURE (*K* = 2). Each genotype is represented by a vertical bar. The colored subsections within each vertical bar indicate membership of the genotype to different origins.

From the STRUCTURE analysis, the most probable number of genetically distinct groups (K) was two (Figure 2A) based on Delta *K*. At the threshold of *Q* = 0.7, 105 samples (38.9%) could be classified into the first genetic cluster, whereas 103 samples (37.0%) could be classified into the second cluster. There were 62 admixed samples (23.0%) that had *Q*-value < 0.7 in either cluster (Figure 2B and Supplementary Table S6). The first cluster comprised 58 accessions from CRIG, 28 from Togo, 18 from Cote d’Ivoire (CNRA), and one from an unknown source. The second cluster comprised all 33 accessions introduced from Vietnam, 27 accessions from CRIG, 10 from Cote d’Ivoire (CNRA), six from Cameroon, 11 from Togo, and 16 from unknown sources (Supplementary Table S6).

**FIGURE 3:**
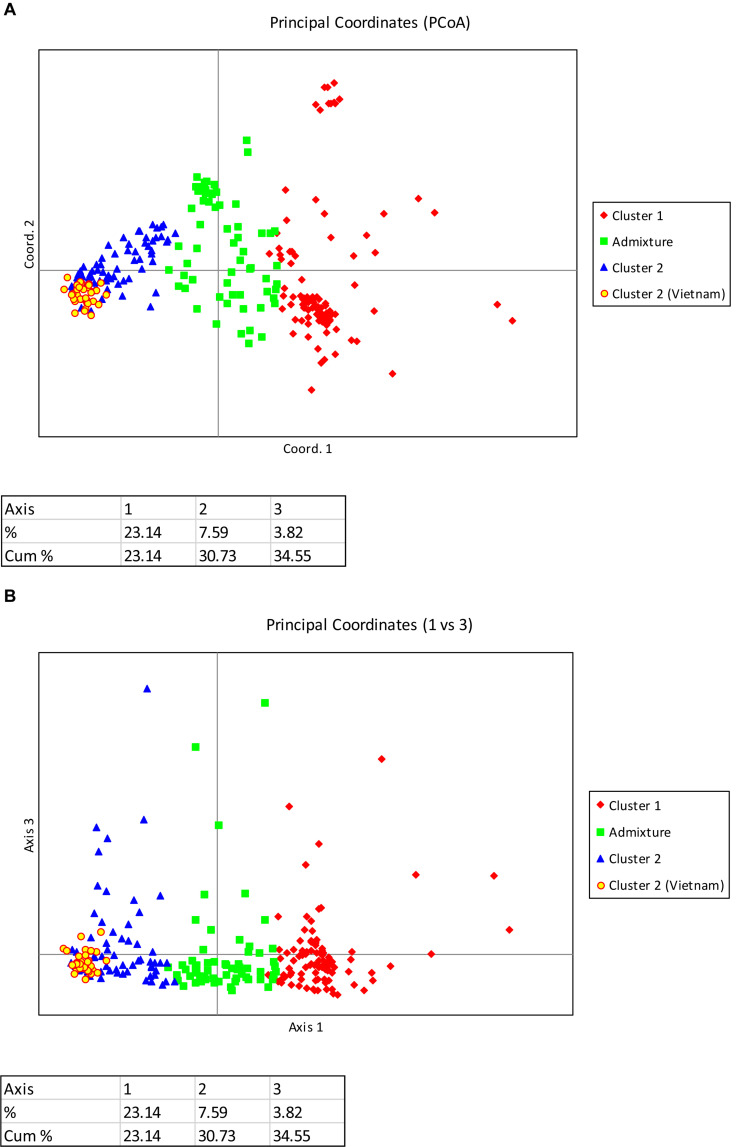
**(A)** PCoA plot of 270 C. *canephora* genotypes in Ghana genotyped at 120 SNP loci and belonging to two main populations plus an admixture group as revealed by STRUCTURE (Axis 1 vs. Axis 2). The admixture group was made up of 62 samples; the first cluster comprised 58 accessions from CRIG, 28 from Togo, 18 from CNRA, and one with unknown sources; the second cluster comprised all 33 accessions introduced from Vietnam, 27 accessions from CRIG, 10 from CNRA, six from Cameroon, 11 from Togo and 16 with unknown sources. **(B)** PCoA plot of 270 C. *canephora* genotypes in Ghana genotyped at 120 SNP loci and belonging to two main populations plus an admixture group as revealed by STRUCTURE (Axis 1 vs. Axis 3). The admixture group was made up of 62 samples; the first cluster comprised 58 accessions from CRIG, 28 from Togo, 18 from CNRA, and one with unknown sources; the second cluster comprised all 33 accessions introduced from Vietnam, 27 accessions from CRIG, 10 from CNRA, six from Cameroon, 11 from Togo and 16 with unknown sources.

**FIGURE 4:**
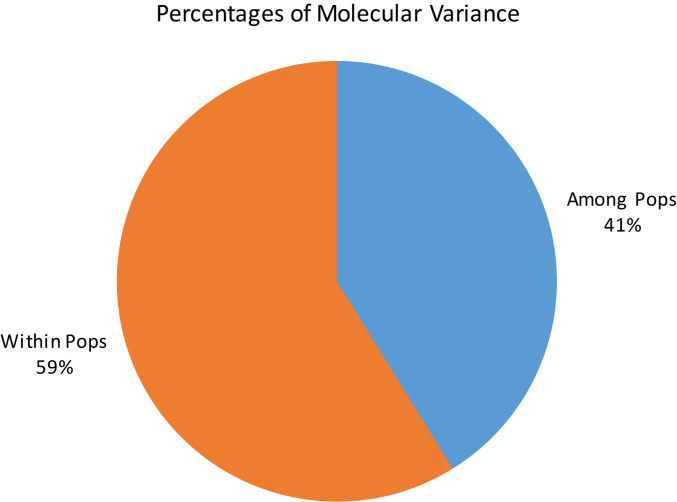
Analysis of molecular variance of *C. canephora* genotypes in Ghana within two main clusters as identified by STRUCTURE.

**FIGURE 5:**
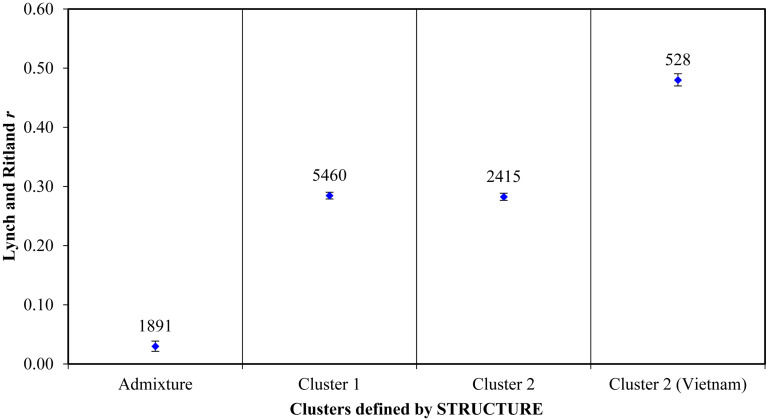
Mean within-population pairwise relatedness based on Lynch and Ritland (1999) index for *C. canephora* genotypes belonging to two main populations plus an admixture group defined by STRUCTURE. Populations as identified by structure: The admixture group was made up of 62 samples; the first cluster comprised 58 accessions from CRIG, 28 from Togo, 18 from CNRA, and one with unknown source; the second cluster comprised all 33 accessions introduced from Vietnam, 27 accessions from CRIG, 10 from CNRA, six from Cameroon, 11 from Togo and 16 with unknown sources. *The mean r value is indicated by the blue diamond shape on each bar. The number of pairs compared per group is indicated above the mean r values. The bars bound the 95% confidence interval about the null hypothesis of ‘no difference’ across the populations as determined by 9999 permutations.*

Principal coordinate analysis based on the results of the STRUCTURE analysis is presented in Figures 3A,B, which provides a complementary illustration of the relationship between the two main clusters. The plane of the first three main axes accounted for 23.1, 7.6, and 3.8% of total variation, respectively. The distinctiveness of the two clusters was clearly revealed. The results of the analysis of molecular variance (AMOVA) provide additional evidence supporting the distinction of the two clusters (Figure 4). The within-population molecular variance accounted for 59.0%, whereas among populations, molecular variance was 41.0%. The interpopulation differentiation was highly significant as shown by Phi-statistics (*P* < 0.001; Excoffier et al., 1992). The Fst value was 0.256 and was highly significant by permutation tests (*P* < 0.001). The distribution of pairwise relatedness (*r*) within clusters was similar for clusters 1 and 2. Trees within the admixture group had the least pairwise relatedness of *r* = 0.03, and the most related pairs were within the Vietnam (cluster 1) group (Figure 5). The associated confidence limits showed that the relatedness values within the two main genetic clusters (1 and 2) were similar and significantly lower and higher than those of the admixture and Vietnam (cluster 1) groups, respectively.

**FIGURE 6:**
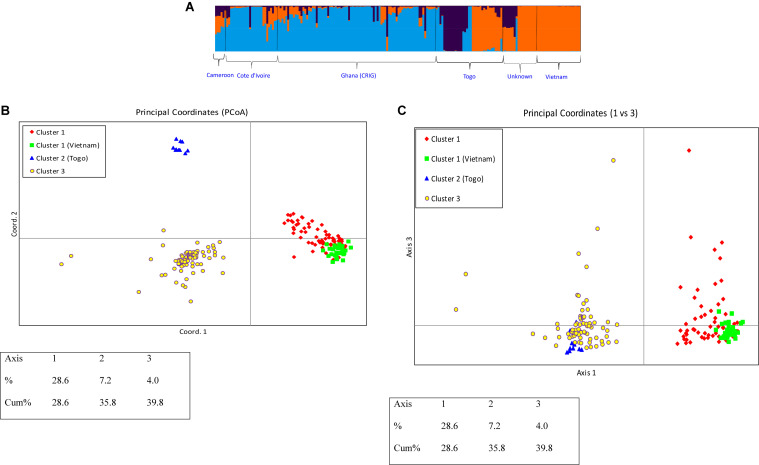
**(A)** *Q*-plot showing clustering of 270 *C. canephora* genotypes based on analysis of genotypic data at 120 SNP loci using STRUCTURE (*K* = 3). Each genotype is represented by a vertical bar. The colored subsections within each vertical bar indicate membership of the genotype to different origins. **(B)** PCoA plot of 270 *C. canephora* genotypes in Ghana genotyped at 120 SNP loci and belonging to three main populations as revealed by STRUCTURE with delta *K* = 3 (Axis 1 vs. Axis 2) **(C)** PCoA plot of 270 *C. canephora* genotypes in Ghana genotyped at 120 SNP loci and belonging to three main populations as revealed by STRUCTURE with delta *K* = 3 (Axis 1 vs. Axis 3).

Because the Delta *K* plot also showed that the next highest delta *K* value is *K* = 3, the studied *C. canephora* germplasm could also be classified into three genetic clusters. Under this scenario, each germplasm source/origin was represented by accessions belonging to the three genetic clusters except germplasms from Vietnam, which all belonged to one genetic cluster (Figure 6A). Additionally, under the *K* = 3 scenario, a new cluster was separated, which exclusively included 10 samples from Togo, and the plane of the first three main axes accounted for 28.6, 7.2, and 4.0% of total variation, respectively (Figures 6B,C).

## Discussion

Germplasm collections of perennial crops remain valuable tools for the improvement of crop species either through direct cultivation of genotypes, backcrossing to introgress important traits, or hybridization to generate new varieties. To maximally utilize such introductions and/or collections, detailed information is required about the population structure of entire germplasm collections in a breeding program as well as the diversity and relatedness available within each source of introduction or collection to inform the choice of parental genotypes in clonal or hybrid variety development. In the present study, we used SNP markers to assess the diversity, population structure, parentage, and labeling errors in *C. canephora* genotypes from different sources over different time periods at CRIG. The implications of mislabeling and population structure in the *C. canephora* breeding program are discussed.

### Identification of Mislabeling

Mislabeling of nursery seedlings in *C. canephora*, as in many other tree crops, is difficult to detect as differences in morphological characteristics are often subtle and may only be visible in mature plants or yield-related traits. A total of 400 trees comprising 294 clones/families introduced and/or collected at different time periods at CRIG were genotyped at 120 SNP loci in the present study. These clones and/or families represent more than 30 years of *C. canephora* coffee-breeding history in Ghana. The main goal of *C. canephora* breeding over the years has been to develop varieties with high yield and better cup quality. To achieve this, hybrid variety development strategies through evaluation of progenies of specific combinations of selected parents have been adopted. Also, development of clonal varieties has been through the evaluation of ortets (progeny selected for clone development) selected from high-yielding families in hybrid trials. The identification of mislabeling in the current study emphasizes how a breeding program could benefit significantly from establishment of measures to prevent mislabeling of trees and pollen contamination to ensure varieties replicate expected field performance. The average PID-sib of this SNP panel was 0.71, which was slightly inferior to the average PID-sib (0.519) among the 40 SSR markers developed for *C. canephora* (Hendre et al., 2008). However, the cumulative PID-sib of the 40 SSRs was 1.22 × 10^–12^, which can be achieved by approximately 76 SNPs from this panel (as shown in Supplementary Table S4).

Of the 400 genotypes analyzed, a total of 18.6% mislabeling, comprising both synonymous (trees with the same SNP profiles but different names) and homonymous (trees with the same name but different SNP profiles) mislabeling, were identified. Mislabeling identified in the present study is not unique to *C. canephora* but has been identified in a variety of clonally propagated crop species, such as eucalyptus (Keil and Griffin, 1994), Sitka spruce (Van de Ven and McNicol, 1995), oil palm (Purba et al., 2000), and cacao (Padi et al., 2015) as well as potato (Huamán et al., 2000) and enset (Negash et al., 2002) crop gene banks. Christopher et al. (1999), using RAPD markers, detected mislabeling of cocoa stands in the International Cocoa Genebank collection in Trinidad (ICG, T) to be as high as 30%. Motilal (2004), in a further analysis of the cocoa clones at the ICG, T, showed that the level of mislabeling among clones at the ICG, T was around 12% using SSR markers.

A majority of the mislabeling identified in the present study is the synonymous type, suggesting erroneous labeling of plants in the nursery prior to field planting or likely from wrong replacement over the course of time of dead stands in the field with plants meant for other plots. Also, the synonymous mislabeling identified could be a result of introduction of the same clone but with different names from the same or different countries at different time periods. This was mainly the case for introductions from Togo. The lack of passport data for these introductions, however, makes verification of this assertion challenging. The homonymous mislabeling, on the other hand, could mainly be due to erroneous labeling of ramets (individual plants developed from one coffee plant) at the nursery before field planting.

Furthermore, synonymous mislabeling was identified in the biclonal seed garden at CRIG, Tafo, where the female parent was largely identified to be a different clone meant for a different biclonal seed garden. This also suggests erroneous labeling in the nursery prior to field planting or replacement over the course of time of dead stands in the field with plants meant for different biclonal seed gardens. In Ghana, hybrid varieties derived from crossing between clones with good specific combining abilities in biclonal seed gardens are recommended for cultivation (Anim-Kwapong, 2012). Therefore, mislabeling would have a negative effect on the productivity of *C. canephora* in farmers’ plantations established using hybrid seed varieties. The effect of erroneous labeling is likely proportional to the level of mislabeled maternal or paternal trees. Obviously, use of mislabeled clones in variety development leads to poor performance of coffee hybrids in farmers’ plantations with consequent effects on farmers’ attitude toward adoption of recommended varieties. The ability, therefore, to have the genetic identity of parental clones verified or authenticated routinely using molecular markers is very likely to be an important requirement for significant investments in coffee breeding and to ensure that expected varietal performance is replicated in farmers’ farms.

It is noteworthy that the synonymous mislabeling (or duplicates) we report here represents the lower bound of the existing duplicates in the collection. This is because we only took account of the fully matched genotypes, whereas the samples with a small number of mismatched loci were not considered as confirmed duplicates. In practical application, because genotyping errors always occur, a threshold of mismatches needs to be developed as reported by Sedlacek et al. (2016). The distribution of genotypic differences between individuals can also be achieved using the method of Kalinowski et al. (2006). As shown by the Supplementary Figures S1A,B, the mismatch distribution of the 400 coffee accessions indicates that two *C. canephora* accessions likely differ by at least six loci in their multilocus SNP profiles. There are 19 near matched groups (including 60 accessions) that have fewer than five mismatched loci and are all likely synonymous groups too. This suggests that 41 accessions (out of the 60) are duplicated accessions and need to be removed from the collection. Nonetheless, repeated genotyping needs to be applied on these near-matched samples to confirm the synonymous mislabeling as was demonstrated on cacao (Zhang et al., 2006).

### Parentage and Pedigree Verification

As stated under the results section, parentage of the larger proportion of coffee seedling progeny were observed as not conforming to breeders’ records. This anomaly may be due to mislabeling, pollen contamination, or extraneous seeds in the seed lot prior to sowing. By far, wrong pollen use or extraneous seeds in the seed lot prior to sowing appear to account the most for the source of pedigree error in the set of progenies analyzed from the controlled manual crosses. This assertion is supported by the predominance of errors associated with the male parents. Usually, skilled manual pollinators carry out a number of crosses each season to generate progenies for evaluation in our *C. canephora* improvement program at CRIG. Therefore, among the set of progenies derived through manual pollinations, the wrong pedigree of some progenies is very likely as a result of errors made by manual pollinators during the pollen-labeling process prior to physical crossing or wrongful labeling of parental trees in the field. Detection of pedigrees that do not match breeders’ records is not unique to our study. Similar to our findings, McIntyre and Jackson (2001) and Corley (2005) detected pedigrees different from breeders’ records in their analyses of sugarcane and oil palm progenies, respectively. Similarly, in recent years, such errors were reported in pine (Grattapaglia et al., 2014) and cacao (Padi et al., 2015). Pruvot-Woehl et al. (2020) in their study on DNA fingerprinting of *C. arabica* varieties showed that, for seeds that have not moved through formal pathways, pollen contamination may have caused genetic drift, which has resulted in much less genetic conformity (39%) in an important Arabica coffee variety, *Geisha*.

For 20 progenies derived from an open-pollinated biclonal seed garden, none had pedigree (both parents) matching that of breeders’ records as expected. Only four had one parent matching expectation with 3 and 1 matching maternal and paternal parent expectation, respectively. A majority of the probable paternal parents identified from the progenies derived from the open pollinated biclonal seed garden were clones from a distant germplasm plot, which was approximately 200 m away. This observation, however, is not surprising as several studies have shown that pollen from coffee could be carried by pollinating agents (wind or insects) over up to a 2 km distance (Berthaud, 1985; Jha and Dick, 2010). This has significant implications for isolation distance in the establishment of *C. canephora* seed gardens, which, in this case, needs to be far more than 200 m away from any coffee planting, particularly that *C. canephora* is predominantly an outcrossing crop. Considering the inherent difficulties in generating hybrid seed through manual pollinations (due to the short window within which flowers are opened and receptive for pollination), biclonal seed gardens are the more practical means for hybrid seed development in *C. canephora*.

The performance of one family analyzed in the current study shows the negative effect of erroneous labeling or pollen contamination on variety development as well as replication of expected variety performance in farmers’ plantations. Two out of six progenies from the intended H234 × H207 cross had the paternal parents wrongly assigned. Both of these parents with pedigrees E139 × E186 (for H234) and A213 × A115 (for H207) were high-yielding ortets (progeny selected for clone development) selected from high-yielding families in previous hybrid trials at CRIG (unpublished data). Given the lack of relationship between the two parental clones (judging from their respective pedigrees), it was expected that they might combine well to give rise to high-yielding progenies. Superior performance was largely observed in the four progenies that had both maternal and paternal parentages correctly assigned compared with the two progenies that had the wrong paternal parents, either MMC26 or A129. In the present study, significant differences were observed in the plots planted with the H234 × H207 family. Our analysis shows that the differences observed were due to the non-uniformity of parentage of the progeny trees in this family. Progeny trees that had the correct parentage (H234 × H207) were more vigorous and yielded 2.6 kg wet cherry/tree more than those that had MMC26 or A129 paternal parentage (through pollen contamination or tree mislabeling). The higher vigor of the progenies that had the correct parentage (H234 × H207) is likely due to the specific combining ability of the parental clones. This early vigor in tree crops has been used as a selection criterion for productive varieties, and parents are, therefore, specifically selected for this trait in tree crop breeding programs (Padi et al., 2017). Similar loss in vigor and yield were reported by Padi et al. (2015), in which progenies of cacao that had the wrong parentage due to pollen contamination were less vigorous and produced a smaller number of pods per tree compared with trees that had the correct parentage.

The number of progenies assessed for the cross (H234 × H207) in the parentage and pedigree verification analysis is small (six); however, the observed yield loss of 2.6 kg wet cherry/tree from the erroneous parentage is suggestive of a negative impact on productivity due to use of mislabeled clones in coffee cultivar development. Also, a possible increase in inbreeding and loss of diversity could be reduced significantly through such routine parentage analysis in breeding programs.

It is important that significant steps are taken to correct erroneous labeling of trees and wrong parentage of progenies to guarantee anticipated advances from investments in *C. canephora* improvement in Ghana. To eliminate this problem in biparental *C. canephora* seed garden establishment, it is important that due diligence at the nursery is done to ensure that generated clones are labeled correctly. Furthermore, replacement of dead stands in the field should be carried out carefully with the same clones as the dead ones. Additionally, to reduce the chance of pollen contamination and its negative impact on agronomic performance of varieties, it is important to incorporate SNP fingerprinting in *C. canephora* breeding programs. Although SSRs for parentage analysis is well established and widely applied because of their hypervariable nature conferring sufficient statistical power, SNP markers have advantages, such as easier automation and scoring, and are, thus, suitable for high-throughput application at a lower cost. As recently reviewed by Flanagan and Jones (2019), a substantial number of studies compared the power of SNPs to microsatellites for parentage analysis, and they concluded that as few as 100–500 SNPs are sufficient to resolve parentage completely in most cases. In managing a coffee seed garden, it is important to constantly train manual pollinators and check their outputs through SNP marker fingerprinting of progenies sampled each season. Moreover, use of much wider isolation distance of seed gardens from other coffee plantings to reduce pollen contamination is particularly recommended. Ensuring that permanent labels are maintained on each unique tree would be effective in the long-term management of seed garden plots as well. Frequent parentage verification of introduced clones at different time points to eliminate duplications in germplasm collections should be included in the management of coffee germplasm collections.

### Population Structure and Relatedness

The delta *K* calculated by Evanno’s method (Evanno et al., 2005) indicated two genetic clusters in the germplasm collection. The core members of the two clusters (assigned at *Q* = 0.70) were highly differentiated (*F*st = 0.256; *P* < 0.001). The significant divergence was further supported by AMOVA, which showed a large proportion of among-population molecular variance (41.0%). The existence of a significant substructure in *C. canephora* germplasm is reported from different studies (Montagnon, 2000; Dussert et al., 2003; Musoli et al., 2009; Gomez et al., 2009; Bikila et al., 2017). However, most previous studies reported more than two populations in their diversity analysis. This discrepancy could be partially explained by the likely different scope and composition of genetic diversity in the CRIG collection. Some populations may not be proportionally represented by a sufficient number of accessions in the CRIG collection and, thus, were not classified as an independent genetic cluster by STRUCTURE (Kalinowski, 2011). Moreover, because the Delta *K* method detects the uppermost hierarchical level of genetic structure, this may lead to underestimating the number of genetic clusters in this collection. For example, as the plot of delta *K* showed, the studied *C. canephora* germplasm might also be classified into 6, 10, or 12 genetic clusters (Figure 2A). Nonetheless, reference wild populations of *C. canephora*, representing the full spectrum of genetic diversity of the primary gene pool of *C. canephora*, need to be included in future studies to improve the understanding of the substructure in the germplasm collection in Ghana. The importance of SNP markers in detecting genetic groupings in germplasm collections in breeding programs is imperative as such genetic analysis provides better genetic information than that derived from phenotypic data, which tends to be influenced by the environment (Sousa et al., 2017).

Determination of relatedness in breeding programs is important for three main reasons: (1) to evaluate the level of relationship due to co-ancestry among individuals of a breeding population, (2) to study and understand the pedigree structure of offspring derived through crossing, and (3) for genomic prediction (Sousa et al., 2019). Lynch and Ritland (1999) pairwise relatedness revealed similar relatedness among the genotypes in the two main clusters (1 and 2). The genotypes within the admixture group were the least related (*r* = 0.03) as revealed by the Lynch and Ritland (1999) pairwise relatedness analysis. Introductions from other countries are more likely to reflect breeding histories (improved germplasm) with selections made for adaptation in their native environments. The introduced germplasm from Vietnam possessed the highest level of within-group relatedness (*r* = 0.48) compared with other clusters. This suggests the materials introduced from Vietnam were full-sibs (siblings that share the same parents) (Lynch and Ritland, 1999) and further supports the homogeneous origin and narrow genetic base of the materials. Introductions from active coffee-breeding programs should be analyzed for genetic relationships to avoid use of closely related individuals as, in some cases, the most productive clones in a breeding program arise from a common parentage. Although there was a low genetic diversity within the introductions from Vietnam, the PCoA analysis revealed a clear distinction of these genotypes from cluster 1 and the admixture group, which consisted of the majority of the local collections and selections made at CRIG over several years of breeding. This presents an opportunity for incorporation of any desirable variants that would be identified from such introductions into the *C. canephora* breeding program in Ghana. In addition to providing an implied measure of diversity, relatedness estimates are tools that help in the selection of breeding lines to reduce inbreeding and inbreeding depression. The least related genotypes, therefore, provide opportunities for selecting a suit of clones as planting material or as parents for generating seedling varieties with minimal risks of inbreeding.

Results of our pilot study demonstrate the effectiveness of using a small SNP panel in gene bank management, varietal identification, and seed garden verification. However, more SNP markers are needed for the selection of an optimum genotyping panel. We are currently validating more SNPs on the same germplasm collection and eventually will select a core set of SNPs and recommend them for the international coffee community. The selection criteria will be based on pairwise LD, MAF, and PIC. Moreover, because a large number of interpopulation (and subpopulation) hybrids and their parental clones are maintained in gene banks (Bramel et al., 2017), ancestry informative markers (AIM) that have distinct frequency differences across populations need to be selected for assigning ancestry of *C. canephora* genotypes.

## Conclusion

Through this pilot study, we demonstrate that SNP markers are effective tools in fingerprinting germplasm collections from different sources to guide germplasm conservation and variety development in a *C. canephora* breeding program. The set of 120 SNP markers used in this study are effective to characterize the existing diversity of *C. canephora* germplasm collections in Ghana, judging from their high statistical significance for individual genotype identification, parentage analysis, and dissection of population structure. The 120 SNPs used is not our final recommendation genotyping panel. We are currently validating more SNPs on the same germplasm collection and eventually will select a genotyping panel for coffee varietal identification. To the best of our knowledge, this study is the first to apply array-based SNP genotyping for molecular characterization of *C. canephora* germplasm collection. The understanding of genetic diversity and population structure in coffee-breeding programs provides opportunities for selection of positive variants for direct cultivation or as parental material for recurrent crossing and further selection.

With a growing demand for improved coffees, authentication of parentage is recognized as an important requirement in *C. canephora* breeding as regards developing improved varieties that would replicate performance in farmers’ fields. To this end, the set of markers reported in the present study are sufficient for assignment of parentage in *C. canephora* given the high total exclusion probability of 99.997 × 10^–2^ obtained from the parentage analysis. The result also suggests that this array-based SNP genotyping can be efficiently used to improve our understanding of the mating system, pollen dispersal, and seed garden quality in *C. canephora*.

This study demonstrates the utility of SNPs to detect mislabeling in a *C. canephora* breeding program that includes germplasm from multiple sources. The observed level of mislabeling in the present study, which is likely underestimated, is comparable to those in other tree crops (as cited earlier for cocoa) that rely on a seed garden system of variety development. This calls for implementation of measures such as training of manual pollinators, field labeling of clones with metal labels, and routine use of SNP markers to fingerprint germplasm collections to eliminate or reduce such erroneous labeling and its effect on variety development in tree crop breeding. Evidence gathered from the present study revealed the negative effect of mislabeling and/or pollen contamination on a practical coffee-breeding program in which unintended crosses are less vigorous and yield less than intended crosses. This reinforces the requirement to incorporate routine DNA fingerprinting to guide the selection of potential parents to reduce inbreeding and increase genetic gain in a *C. canephora* improvement program.

## Data Availability Statement

The original contributions presented in the study are included in the article/Supplementary Material, further inquiries can be directed to the corresponding author/s.

## Author Contributions

AA, FP, and DZ conceived the experiment. AA, FP, DZ, and LM conducted the experiment, analyzed the data, and wrote the manuscript. All the authors contributed to the article and approved the submitted version.

## Conflict of Interest

The authors declare that the research was conducted in the absence of any commercial or financial relationships that could be construed as a potential conflict of interest.

## Footnotes

1 http://taylor0.biology.ucla.edu/structureHarvester/
