# Supplementary material for: Effectiveness of Single Nucleotide Polymorphism Markers in Genotyping Germplasm Collections of Coffea canephora Using KASP Assay
Source: Front Plant Sci. 2021 Jan 25;11:612593. doi: 10.3389/fpls.2020.612593 (PMC7868401; doi:10.3389/fpls.2020.612593)
Supplement: Supplementary file 2 [file Image_1.docx]

**Supplementary Figure S1 (A)** Mismatch distribution of 400 *C. canephora* accessions in the CRIG *C. canephora* collection, based on 120 SNP markers.

**Supplementary Figure S1 (B)** Mismatch distribution of 400 *C. canephora* accessions in the CRIG *C. canephora*, based on 120 SNP markers, highlighting up to 30 mismatched loci where the first five mismatches were likely caused by genotyping error rather than true genotypic difference.
